# 

**Published:** 1894-09

**Authors:** 


### CONTENTS.

ORIGINAL COMMUNICATIONS—

Two Major Amputations. By Hunter P. Cooper, M.D., Attending Surgeon, Grady Hospital, Atlanta, Ga. .... 385

A Case of Morphine Poisoning Treated with Permanganate of Potash. By J. I. Darby, M.D., Americus, Ga. .... 389

Conservatism in Abdominal and Pelvic Surgery. By J. C. Olmsted, M.D., Atlanta, Ga. .... 392

Conservatism in Abdominal and Pelvic Surgery. By Floyd W. McRae, M.D., Atlanta, Ga. .... 395

Case Report—Extra-Uterine Superfetation. By R. R. Kime, M.D., Atlanta, Ga. 399

Scopolamine Hydrobromate. By Arthur G. Hobbs, M.D., Atlanta, Ga. .... 401

Asexualization for the Prevention of Crime and the Curtailment of the Propagation of Criminals. By F. L. Sim, M.D., Memphis, Tenn. .... 403

SOCIETY REPORTS—

Atlanta Society of Medicine .... 407

CORRESPONDENCE—

Watermelon Seed in Windpipe. Tracheotomy. Recovery .... 414

EDITORIAL—

Treatment of Epilepsy and Chorea by Partial Tenotomies of the External Muscles of the Eyes .... 416

One Board or Three? .... 419

Our "Society Reports" .... 420

Wants to Study Medicine .... 421

The Bedford Mineral Springs Co .... 421

SELECTIONS AND ABSTRACTS—

General Medicine and Therapeutics .... 422

Obstetrics and Gynecology .... 431

Skin Diseases and Syphilis .... 435

Surgery .... 441

MEDICAL ITEMS .... 444

BOOK REVIEWS .... 446

ADVERTISERS' INDEX TO ADVERTISEMENTS .... xii

CLASSIFIED INDEX TO ADVERTISEMENTS .... xxxvii

ITEMS OF INTEREST .... x, xi

